# **Examining the immunological responses to COVID-19 vaccination in multiple myeloma patients: a systematic review and meta-analysis**

**DOI:** 10.1186/s12877-024-05006-0

**Published:** 2024-05-08

**Authors:** Hamid Harandi, Parisa Fallahtafti, Amirali Karimi, Seyedeh Melika Hashemi, Mehrdad Mahalleh, Moein Ashouri, Mohammad Amin Salehi, Armin Hoveidaei

**Affiliations:** 1 Research Center for Antibiotic Stewardship and Antimicrobial Resistance, Imam Khomeini Hospital Complex, Tehran University of Medical Sciences, Tehran, Iran; 2 School of Medicine, Tehran University of Medical Sciences, Tehran, Iran; 3 Uro-Oncology Research Center, Tehran University of Medical Sciences, Tehran, Iran; 4 Student Research Committee, Shahid Beheshti University of Medical Sciences, Tehran, Iran; 5 Rheumatology Research Center, Tehran University of Medical Sciences, Tehran, Iran; 6 Tehran Heart Center, Cardiovascular Diseases Research Institute, Tehran University of Medical Sciences, Tehran, Iran

**Keywords:** COVID-19, Multiple myeloma, Plasma cell dyscrasia, SARS-CoV-2, Vaccine

## Abstract

**Background:**

Impaired immune response in multiple myeloma renders the patients vulnerable to infections, such as COVID-19, and may cause worse response to vaccines. Researchers should analyze this issue to enable the planning for special preventive measures, such as increased booster doses. Therefore, this meta-analysis aimed to evaluate the response and efficacy of COVID-19 vaccines in patients with multiple myeloma.

**Methods:**

This meta-analysis followed PRISMA 2020 guidelines, conducting a comprehensive database search using specified keywords. Study selection involved a two-phase title/abstract and full-text screening process. Data extraction was performed by two researchers, and statistical analysis involved meta-analysis, subgroup analysis based on vaccine dosage and study time, random effects meta-regression, and heterogeneity testing using the Q test.

**Results:**

The meta-analysis revealed that patients with multiple myeloma (MM) had a lower likelihood of developing detectable antibodies after COVID-19 vaccination compared to healthy controls (Log odds ratio with 95% CI: -3.34 [-4.08, -2.60]). The analysis of antibody response after different doses showed consistent lower seropositivity in MM patients (after first dose: -2.09, [-3.49, -0.69], second: -3.80, 95%CI [-4.71, -3.01], a booster dose: -3.03, [-5.91, -0.15]). However, there was no significant difference in the mean level of anti-S antibodies between MM patients and controls (Cohen’s d -0.72, [-1.86, 0.43]). Evaluation of T-cell responses indicated diminished T-cell-mediated immunity in MM patients compared to controls. Seven studies reported clinical response, with breakthrough infections observed in vaccinated MM patients.

**Conclusions:**

These findings highlight the impaired humoral and cellular immune responses in MM patients after COVID-19 vaccination, suggesting the need for further investigation and potential interventions.

**Supplementary Information:**

The online version contains supplementary material available at 10.1186/s12877-024-05006-0.

## Introduction

Multiple myeloma (MM) patients are notably vulnerable to viral and bacterial infections [1]. Data from a comprehensive population-based study indicate that these patients have a 7-fold higher risk for bacterial and a 10-fold increased risk for viral infections [1]. Additionally, the study reveals that approximately 22% of deaths among all MM patients documented in the nationwide Swedish Cancer Registry at one year of follow-up were attributed to infections [1]. Various immune effector mechanisms are compromised due to their disease in MM patients even before the initiation of antimyeloma therapy [2]. A survey reported that about half of these patients experience one or more periods of infection in the year preceding antimyeloma therapy, with 43% experiencing infections in the first six months following therapy initiation [3]. Conversely, the risk of inpatient mortality due to COVID-19 was approximately 34% among adult patients with hematological malignancies [4]. These findings accented to the necessity of urgent action to identify preventive treatment options for these patients. Vaccination has emerged as one of the most successful preventive interventions against infections, saving millions of lives. However, both Myeloma itself and antimyeloma therapy can reduce immune competence and impair the development of long-term immunological memory. This issue poses a significant obstacle to effective vaccination in patients with MM [2]. A study involving 52 MM patients and their response to vaccination against influenza, S. pneumoniae and Haemophilus influenzae type b (Hib) revealed that only 19% of MM patients could develop effective antibody titers to all three strains of vaccine and 10% against two viral strains of the vaccine [5]. Another study in 2015 had better results showing that 9–19% of patients already had sufficient antibody titers against at least one strain of influenza virus. This number increased by 20–40% after a single dose vaccination and doubled after the second boost [6]. Novel vaccines even show better performances in hematologic malignancies. 80.2% of these patients could express sufficient humoral response in a trial with two doses of the new adjuvanted recombinant varicella zoster virus glycoprotein E vaccine [7]. Vaccination against Hepatitis B is also recommended in MM patients who live in or travel to areas endemic for hepatitis B or patients with sexual partners with chronic hepatitis B infection [8]. Vaccination against several bacterial agents like Pneumococci, Hemophilus influenzae, and Meningococci has also been studied and recommended in MM patients [9–12].

Vaccination against SARS-CoV-2 constitutes a major preventive option, especially for vulnerable patients [13]. However, patients with MM were left out from most SARSCoV-2 mRNA vaccine trials, resulting in limited information regarding the safety and efficacy of vaccines in this population [2]. Available data demonstrate that the antibody response provoked by COVID-19 in patients with hematological malignancies against SARS-CoV-2 is ineffective [14]. As COVID-19 persists in increasing the morbidity and mortality rate in these patients, synthesizing evidence to inform decision-making and provide recommendations becomes imperative. Consequently, we aimed to run a meta-analysis to evaluate the antibody response, and efficacy following vaccination against SARS-CoV-2 in patients with MM.

## Methods

### Overview and database search

This meta-analysis followed the PRISMA (Preferred Reporting Items for Systematic Reviews and Meta-Analyses) 2020 guidelines. First, we searched the databases of PubMed, Embase, Web of Science, and Cochrane on August 19th, 2022. Keywords for “Multiple Myeloma”, “COVID-19”, and “Vaccination” were chosen from the related previous studies and medical subject headings (MeSH) website to build the search strategy. All the keywords were searched as title/abstract/keywords in the databases. Supplementary Table 1 contains the search terms for each database.

### Study selection

All the records were downloaded into EndNote software and the duplicates were removed both by the application and manually. Then the records were uploaded to the Covidence database and duplicates were once again removed by the website. The records then underwent a two-phase screening process. First, they were screened based on their title and abstract. The approved records were then screened by their full texts and the eligible studies were included in this meta-analysis. Whenever any disagreements arose between the two researchers involved in the screening process (A.K. & H.H.), they discussed the matter to solve the problem. If disagreements remained, they sought another independent opinion for final decision.

A researcher (A.K.) went through the citations of the included articles to find any possible suitable records manually. These manual records then entered the full text screening and they were combined with eligible studies through database searching to determine the overall included studies.

### Inclusion/exclusion criteria

We included all the original English studies that compared response to COVID-19 vaccines in patients with MM compared to healthy controls. If the study did not specifically report the patients with MM, but rather reported the data for patients with plasma cell disorders as a whole without individual data for MM subgroup, we included them to avoid data loss only if the percentage of patients with MM were 85% or more of the overall patients. To eliminate the bias related to the inclusion of research involving patients with other plasma cell disorders (PCD) besides MM, we conducted a meta-regression analysis to assess the impact of incorporating these studies. Therefore, the exclusion criteria were the following:

1. No MM groups.
2. Not original, i.e., reviews or commentaries.
3. Case reports and case series.
4. No healthy control groups.
5. No vaccines, or vaccines other than COVID-19.
6. Non-human studies.
7. Same settings published elsewhere; this is particularly true when similar authors report the updates of similar patients in a future study, in such case, we included the later study.
8. Abstracts or studies without full texts.
9. Non-English studies.

### Data extraction

We designed an excel sheet before data extraction. Two researchers extracted the data of the included studies into the excel sheet (P.F.T. & M.H.), and two other independent researchers (H.H. & A.K.) rechecked the extracted data for validity. After checking the validity of the extracted data, it was used for the synthesis of the systematic review and meta-analysis.

The data extraction excel sheet contained the following information:

Study title, first author, country, year, study design, controls and matching status, types of assessed vaccines, doses received, characteristics of cases and controls, including their numbers, mean age (SD), disease status, and treatments received, anti-spike antibody responses in cases and controls, including the criteria used for response, mean (SD) antibody titers, number of positive humoral responses to vaccine, and any other subgroups that antibody responses are reported for, cellular immunity response in cases and controls, including response criteria, mean (SD) T-cell response based on the criteria, number of patients with positive response, other cellular immunity components than T-cells measured, and any other subgroups that cellular immunity responses are reported for, and finally, clinical response to the vaccines in cases and controls, including, number of COVID-19 infections, severe COVID-19 cases, deaths, hospitalization, ICU admission, and any other clinical criteria for any other subgroups mentioned in the studies.

### Statistical analysis

The statistical analysis in this study involved conducting a meta-analysis using Stata version 17. The analysis was performed on two different groups of studies. The first group included studies that measured the AB concentration separately in cases and controls, while the second group included studies that reported the number of AB-positive and AB-negative patients.

Subgroup analysis was then conducted based on the dosage of the vaccine and the study time after vaccine injection. This allowed for the examination of potential differences in outcomes based on these factors.

To further explore the factors influencing the outcomes, random effects meta-regression was performed. The meta-regression analysis included three factors: vaccine type, time after injection, and whether the study focused on MM or PCD. These factors were considered as potential sources of heterogeneity in the meta-analysis.

To assess the heterogeneity and test for group differences among the included studies, the Q test was used. The Q test helps to evaluate whether the observed variations among the effect sizes across the studies are due to chance or represent genuine differences.

### Quality assessment

We used the Newcastle-Ottawa scale (NOS) to assess the quality of the included studies [15]. The total score is out of nine, and we considered a study of poor quality if they could not receive at least four.

## Results

### Study selection

**Fig. 1:**
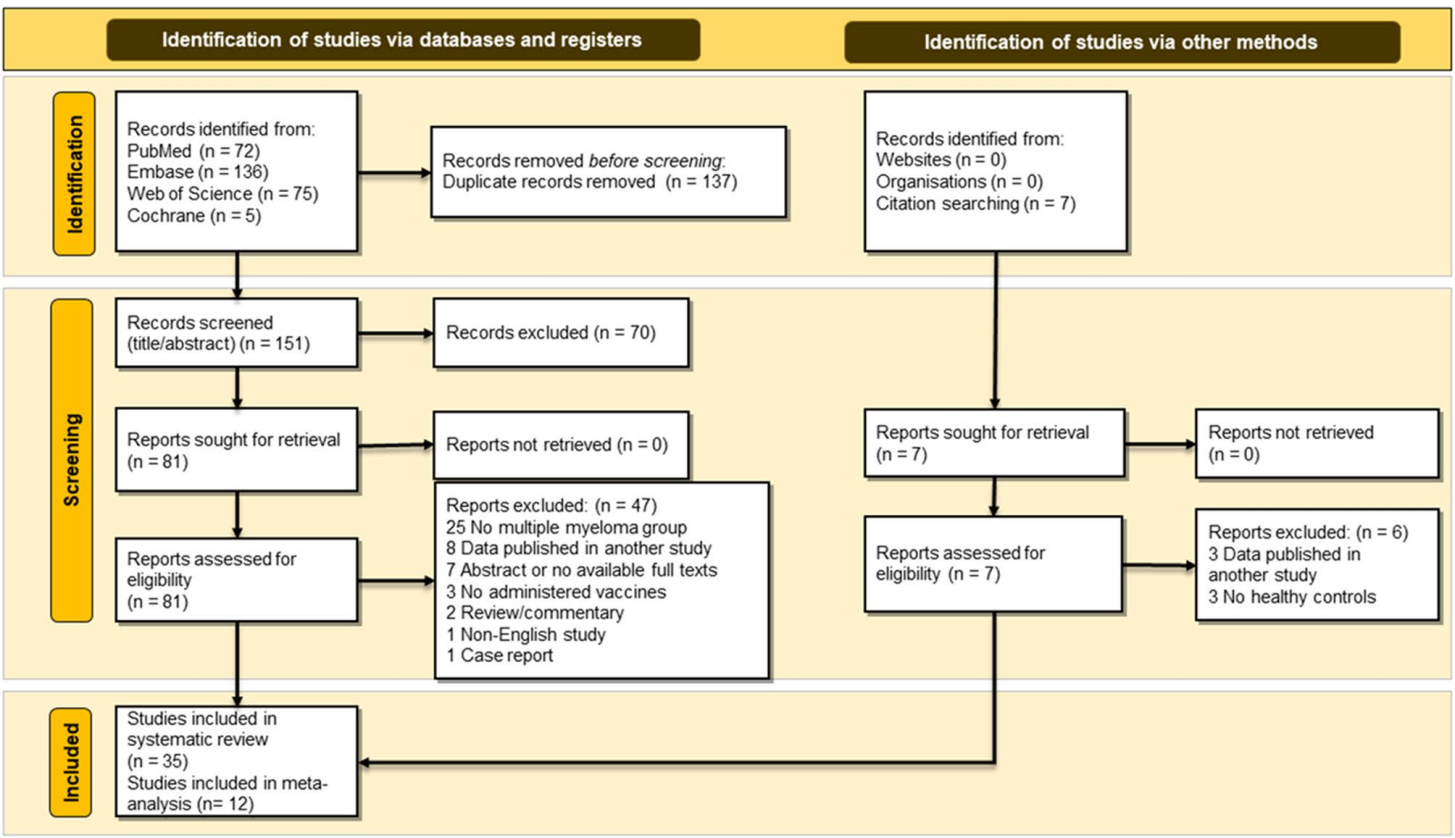
PRISMA 2020 flow diagram of the study selection process

Our systematic search strategy yielded 288 relevant studies, of which 137 were duplicates. Then, a total of 70 studies were excluded after screening through title and abstract. We reviewed the full text of 81 studies, and 47 were excluded for the reasons demonstrated in Fig. 1. Finally, 35 studies were eligible for inclusion, and 12 studies were included in the meta-analysis. The flow diagram is shown in Fig. 1.

### Quality assessment

The results of the quality assessment of the included studies are shown in Supplementary Table 2. The quality assessment was based on selection, comparability of cases and controls based on the design or analysis, and exposure. All included studies had sufficient quality with quality score ranging from 4 to 9.

### Study characteristics

The included studies were conducted in either 2021 or 2022. Most of the studies were conducted in Europe (24), followed by the United States (13), while the least number of studies (4) were conducted in Asia. Thirty-four of the included studies were cohort studies; the remainders were three case-control studies and one cross-sectional study. Seventeen studies included healthy participants as controls. A total of 13,274 patients with a confirmed diagnosis of MM and 509,844 healthy controls were included in the selected studies. The median age of MM patients ranged from 62.9 to 83.0 years. All of the participants received either one or two doses of the following vaccines Pfizer (BNT162b2), AstraZeneca (ChAdOx1 nCoV-19), Moderna (mRNA-1273), or Janssen/Johnson & Johnson (Ad.26.COV2). Additionally, three and one studies evaluated vaccine response after the third and the fourth dose of the Pfizer or Moderna vaccine, respectively. Most selected studies evaluated B-cell response after vaccination, while only six considered T-cell response after vaccination. Also, six studies measured antibodies against COVID-19 prior to vaccination. More detailed characteristics of the included studies in this systematic review are outlined in Table 1.

**Table 1:** Characteristics of the included studies

Author/Year	Country	Study Design	Matching	No. of PCD group	% of MM among PCD group	Male No. (%) of MM	Mean (SD or range) age of PCDs	No. of healthy controls	Male No. (%) of controls	Mean (SD or range) age of controls	Type of assessed vaccines	Other characteristics of MM (Disease status, treatment, etc.)
Abdallah 2022 [16]	USA	Cohort	-	149	87.91%	76 (51)	69 (40–95)	-	-	-	2xModerna or 2xPfizer	10 (8%) newly diagnosed on induction therapy/ 46 (35%) on maintenance therapy/ 75 (57%) relapsed or refractory MM, 66 (51%) monotherapy/ 65 (49%) combination therapy / 101 (77%) high dose chemotherapy and autologous stem cell transplant / 11 previously infected with COVID
Abella 2022 [17]	Spain	Cross-sectional	Age, sex, and time after complete vaccination schedule	10	100%	5 (50)	74 (68–79)	36	-	62 (48–77)	2xModerna or 2xPfizer	100% receiving treatment
Bird 2021 [18]	United Kingdom	Cohort	-	93	100%	55(59.1)	67 (59–73)	-	-	-	1xPfizer (51.6%) or 1xAstraZeneca (48.4%)	48 (52%) complete response or very good partial response / 16 (17%) partial response /27 (29%) patients with stable or progressive disease, therapies: (66.7%) immunomodulator/ (27.3%) PI / (63.6%) Steroid/ (31.8%) Anti-CD38 antibody / (15.2%) Other // IgG 61 (65.6), IgA 21 (22.6), LC (lambda or kappa) 9 (9.68), Other 2 (2.15)
Bitoun 2021 [19]	France	Case Control	-	27	100%	12(44)	-	28	8 (28)	58 (median)	2xPfizer	17 (62%) receiving treatment/ 9 Daratumumab-based / 18 non-Daratumumab-based
Chan 2022 [20]	United Kingdom	Cohort	-	188	82.44%	-	-	-	-	-	2xPfizer (69%) 2xChAdOx1 (25%) unknown (6%)	
Chung 2021 [21]	USA	Cohort	-	221	100%	114 (51.6)	65 (34–85)	69	15 (21.7)	31 (22–67)	**Control**: 2xBNT162b2 (84.1%), 2xmRNA1273 (15.9%) **MM**: 2xBNT162b2 (77.8%), 2xmRNA-1273 49 (22.2%)	209 (94.6%) receiving treatment/ **Multiple myeloma subtype**: Smoldering 10(4.5) Active 211(95.5)
Enssle	Germany	Case control	Age	77	100%	42 (54.5)	67 (60–72)	24	13 (54.2)	66 (50.25–77.50)	2xBNT162b2	**MM Type**: IgG 37 (48.1), IgA 19 (24.7), LC 16 (20.8), Non-secretory 1 (1.3), smoldering MM 4 (5.2) / **ISS**: 1: 31 (40.3), 2: 19 (24.7), 3: 17 (22.1), NA 10 (13.0)/ **Revised ISS**: 1 23 (29.9), 2 28 (36.4), 3 9 (11.7), NA 17 (22.1) / **High-risk**: no 45 (58.4), yes 18 (23.4), NA 14 (18.2)/ **Remission status**: CR 30 (39.0), VGPR 17 (22.1), PR 9 (11.7), SD 5 (6.5), PD 14 (18.2), NA 2 (2.6) / **Previous SARS-CoV-2 infection**: 0 (0.0)
Fattizzo 2022 [22]	Italy	Cohort	-	110	97.27%	-	-	-	-	-	2xPfizer 2xModerna	
Fillmore 2021	USA	Cohort	-	6891	100%	-	-	-	-	-	-	
Gavriatopoulou 2022	Greece	Cohort	-	35	82.85%	16 (45.7)	66 (74)	-	-		2xPfizer	**Remission status**: 10 (34.5%) in SCR/CR / 4 (13.8%) VGPR / 11 (37.9%) PR / 1(3.5%) MR/SD / 1 (3 5%) PD
Ghandili 2021	Germany	Cohort	-	82	95.12%	-	67.5 (40–85)	78	45 (58)	51.3 (7.5)	Pfizer or Moderna or AZD1222 (AstraZeneca)	
Ghandili 2021 [23]	Germany	Case control	Sex, vaccine type	49	100%	49 (59.8)	-	-	-	-	-	38 (45.1%) anti-CD38-targeting/ 2 (2.4%) anti-SLAMF7 / 52 (63.4%) immunomodulatory drug / 13 patients (15.9%) under active surveillance without current treatment / 13 (15.9%) quadruplet treatment / 75.6% in deep remission (≥ VGPR)
Giuseppe 2022 [24]	Italy	Case control	Age, Sex	127	100%	71 (55.9)	69.5 (45–85)	50	-	-	2xPfizer	**Therapy type**:DRd 29 (44.6%), VTd 8 (12.3%), DVd 7 (10.8%), KRd 6 (9.2%), Kd 4 (6.2%), R Maintenance 4 (6.2%), VMP 3 (4.7%), EloRd 3 (4.7%), Belantamab Mafodotin motherapy 1 (1.5%) / **Time from diagnosis**: Newly Diagnosed 63 (49.6), Refractory/Relapsed Multiple
Greenberg 2021 [25]	USA	Cohort	-	44	100%	14 (32)	64 (57–69)	-	-	-	2xPfizer (50%) or Moderna (50%)	27 (61%) on therapy/ 17 (39%) not on therapy / lenalidomide (39%), daratumumab (16%), pomalidomide (9%)
Gung 2022 [26]	USA	Cohort	-	66	62.12%	-	-	-	-	-	2xPfizer or 2xModerna or 1xJ&J	18 autologous stem cell transplantation/ most myeloma patients were on active treatment. The patients with MGUS, SM, and amyloidosis were off treatment. / Myeloma off treatment 28 Myeloma on-treatment 38
Haggenburg 2022 [27]	Netherlands	Cohort	-	186	100%	117 (62.9)	69.86	28	-	-	2xModerna	First-line therapy (28) / Daratumumab (52) / IMiDs (55) / <9 months after autologous HCT (51)
Haggenburg 2022 [28]	Netherlands	Prospective Observational Cohort	Age	157	100%	-	-	37	12 (32)	57 (10)	2xmRNA-1273	First-line therapy: 23, Daratumumab-containing therapy: 44, IMiDs 46, < 9 months After autologous HCT (HDM) 4
Hallmeyer 2022 [29]	USA	Cohort	-	76	100%	-	-	-	-	-	3xPfizer or 3xModerna	-
Henriquez	France	Cohort	-	72	100%	37 (57)	-	23	-	53 (25–73)	2xPfizer	11 had a history of previous SARS-CoV-2 infection. At the time of vaccination, 48 patients were being treated with an anti-CD38 immunotherapy-based regimen (anti-CD38 group), and 24 patients were not being treated (non–anti-CD38 group)
Marasco 2022 [30]	Italy	Prospective Observational Cohort	Age, Sex	52	100%	-	-	163	-	-	2xmRNA-1273 or 2xBNT162b2	-
Nooka 2022 [31]	USA	Cohort	-	238	100	124	67.28 (38.44–90.08)	-	-	-	Pfizer (144) Moderna (84) Ad.26.COV2 (4)	-
Ntanasis-Stathopoulos 2022	Greece	Cohort	-	201	100%	114 (56.7)	67 (15)	-	-	-	4xPfizer	43 patients anti-CD38 monoclonal antibodies, 21 patients anti-BCMA antibodies, 137 patients receiving combinations based on PIs and IMiDs
Pimpinelli 2021 [32]	Italy	Prospective Observational Cohort	-	42	100%	23 (54.77)	73 (47–78)	36	18 (50)	81 (79–87)	2xBNT162b2	**Time (months) from beginning of ongoing therapy to vaccination**: median (range): 9 (1–111) / **Ongoing treatments**: PI-based 9(21.4), VTD 2 (4.76), VMP 2 (4.76), VCD 1 (2.38), IRD 2 (4.76), KRD 1 (2.38), PVD 1 (2.38), Daratumumab-based DRD 14 (33.33), Imids-based 19 (45.24), Lenalidomide + dex 17 (40.48), Pomalidomide + dex 1 (2.38), ERD 1 (2.38)
Ramasamy 2021 [33]	United Kingdom	Cohort	-	109	100%	67	62.9 (9.9)	-	-	-	1xPfizer (41.9%) or 1xAstraZeneca (58.1%) vaccinated with 2nd dose(4.6%)	-
Re 2022 [34]	France	Cohort	-	16	100%	-	-	-	-	-	3xPfizer	-
Schiller Salton 2021	Israel	Cohort	sex, age	186	100%	103 (58.8)	67.8 (9.9)	360	-	-	2xPfizer	11 with newly diagnosed MM, 168 with MM, and 8 with AL amyloid / 23 patients (13%) were treated with IVIg
Stampfer 2021 [35]	USA	Prospective Observational Cohort	Age	103	100%	61 (59.22)	68 (35–88)	31	12 (38.7)	61 (26–85)	2xmRNA-1273 or 2xBNT162b2	**Disease status**: active MM 96, smoldering 7 / **Race**: (Caucasian/AAd/Hispanic/Asian/Mee) 75/7/5/8/8 **Treatment regimen (%)**: Proteasome inhibitor (PI) 45 (44), Immunomodulatory agents (IA) 39 (38), PI + IA 11 (11), Antibodies 19 (18), Alkylating agents 3 (3), Other chemotherapy 25 (24), Steroids 87 (84)/ MM type: IgA MM 18, IgG MM 48, Light chain MM 24, Other MM 6, SMM 7
Storti 2022	Italy	Cohort	-	40	100%	8 (21)	-	-	-	-	2xPfizer and 1xModerna (as booster in 16 patients)	6 MGUS, 10 SMM and 24 MM patients
Terao 2022 [36]	Japan	Case Control	Age	206	100%	93 (45.1)	74 (68–79)	94	45 (47.87)	73 (69–78)	BNT162b2	**Time from diagnosis to vaccination**: months (median, IQR) 45.8 (24.1–86.8) / **ISS**: Stage I 69 (34.5), Stage II 65 (32.5), Stage III 66 (33.0) / **Ig type**: IgG 107, IgA 48, IgM 0, IgD 1, Light-chain 49, Non-secretory 1 / **High-risk cytogenetics at diagnosis** (*n* = 171) 36 (21.1) / Absolute lymphocyte count, /µL (IQR): 1275 (913–1694) / Receiving treatments within 6 months before 1st vaccination 171 (83.0)/ Treatment before 1st vaccination (within 6 months): Bd 3, VRd 2, VMP 2, KRd 2, Kd 12/ Lines of therapy, median (IQR): 4 (3–5) / IVIg before and after vaccination 25 (12.1)/ Vaccination with BNT162b2 195 (94.7)
Terao 2022 [37]	Japan	Case Control	-	54	100%	23 (38.3)	75 (47–95)	-	-	-	2xPfizer (59 patients) 2xModerna (1 patient)	**Heavy-chain type**, n (%): IgG 34 (56.7), IgA 17 (28.3), Light-chain only 7 (11.7), Others 2 (3.3)/ Light-chain type, kappa, n (%): 38 (63.3)/ **ISS**, Stage III, n (%): 32 (59.3)/ Absolute lymphocyte count, /µl, median (range): 1281 (468–4896)/ polyclonal IgG, g/l, median (range): 6.28 (2.49–26.31)/ **Time from diagnosis to vaccination**, months, median (range): 42.8 (0–200)/ **Treatment at second vaccination**, n (%): DVd 2 (3.3), DRd 10 (16.7), Dara monotherapy 6 (10.0), IsaPd 6 (10.0), Isa monotherapy 1 (1.7), ERd 1 (1.7), EPd 5 (8.3), VRd 3 (5.0), IRd 5 (8.3), Rd 1 (1.7), Pd 3 (5.0), Iberdomide and dexamethasone 1 (1.7), VMP 2 (3.3), Kd 2 (3.3), Off-treatment 12 (20.0)
Terpos 2021 [38]	Greece	Prospective Observational Cohort	Age, Sex	48	100%	29	83 (59–92)	104	57	83 (65–95)	1xBNT162b2	**MM type**: Smoldering myeloma 9 (18.7%), active myeloma 39 (81.2%)
Thompson 2022 [39]	USA	Retrospective Cohort	-	66	100%	-	-	-	-	-	Third dose of Moderna or Pfizer	-
Wagner	Austria	Case Control	-	70	100%	36 (55.7)	66.5 (8)	66	33 (50)	46.1 (15.1)	**MM** 2xModerna (31.4%) 2xPfizer (68.6%) **Control** 2xModerna (92.4%) 2xPfizer (7.6%)	64.3% with Ongoing immunosuppressive/ immunomodulator treatment. 45.7% Stem cell transplant.
Wang	USA	Cohort	-	1186	100%	609 (51.4)	68.1 (11.6)	508,457	-	-	2xModerna(22%) 2xPfizer(77.1%) 1xJJ(0.9%)	26.6% stem cell transplant/ 59.9% chemotherapy/ 50.2% Targeted therapy/ 12.1% Radiation.
Zaleska	Poland	Cohort	-	60	100%	30 (50)	64 (35–81)	-	-	-	2xPfizer or 2xModerna	**ISS stage system**: ISS I 30 (50%), ISS II 16 (27%), ISS III 8 (13%), No data 8 (13%) / **Disease status**: Untreated 2 (3%), In treatment 39 (65%), In remission 9 (15%), No data 10 (17%) / **Anti-SARS-CoV-2 antibodies at baseline**: 22 (37%)

*Abbreviations* PCD: Plasma cell dyscrasias; SD: standard deviation; No: number; MM: multiple myeloma; PI: Proteasome Inhibitor; LC: light chain; ISS: international staging system; CR: Complete Remission; VGPR: Very Good Partial Response; PR: Partial Response; SD: Stable Disease; PD: Progressive Disease; NA: Not Available; DRd: Daratumumab, Lenalidomide, and Dexamethasone; VTd: Bortezomib, Thalidomide, and Dexamethasone; DVd: Daratumumab, Bortezomib, and Dexamethasone; KRd: Carfilzomib, Lenalidomide, and Dexamethasone; Kd: Carfilzomib and Dexamethasone; R Maintenance: Lenalidomide Maintenance Therapy; VMP: Bortezomib, Melphalan, and Prednisone; EloRd: Elotuzumab, Lenalidomide, and Dexamethasone; MGUS: Monoclonal Gammopathy of Undetermined Significance; SM: Systemic Mastocytosis; HCT: Hematopoietic Cell Transplantation; IMiDs: Immunomodulatory Drugs; HDM: High-Dose Melphalan; ERD: Elotuzumab, Lenalidomide, and Dexamethasone; dex: Dexamethasone; IVIg: Intravenous Immunoglobulin; AAd: African American or African Descent; Mee: Middle Eastern; IQR: Inter-quartile Range

### B-cell response

#### Percentage of AB positive and negative patients

**Fig. 2:**
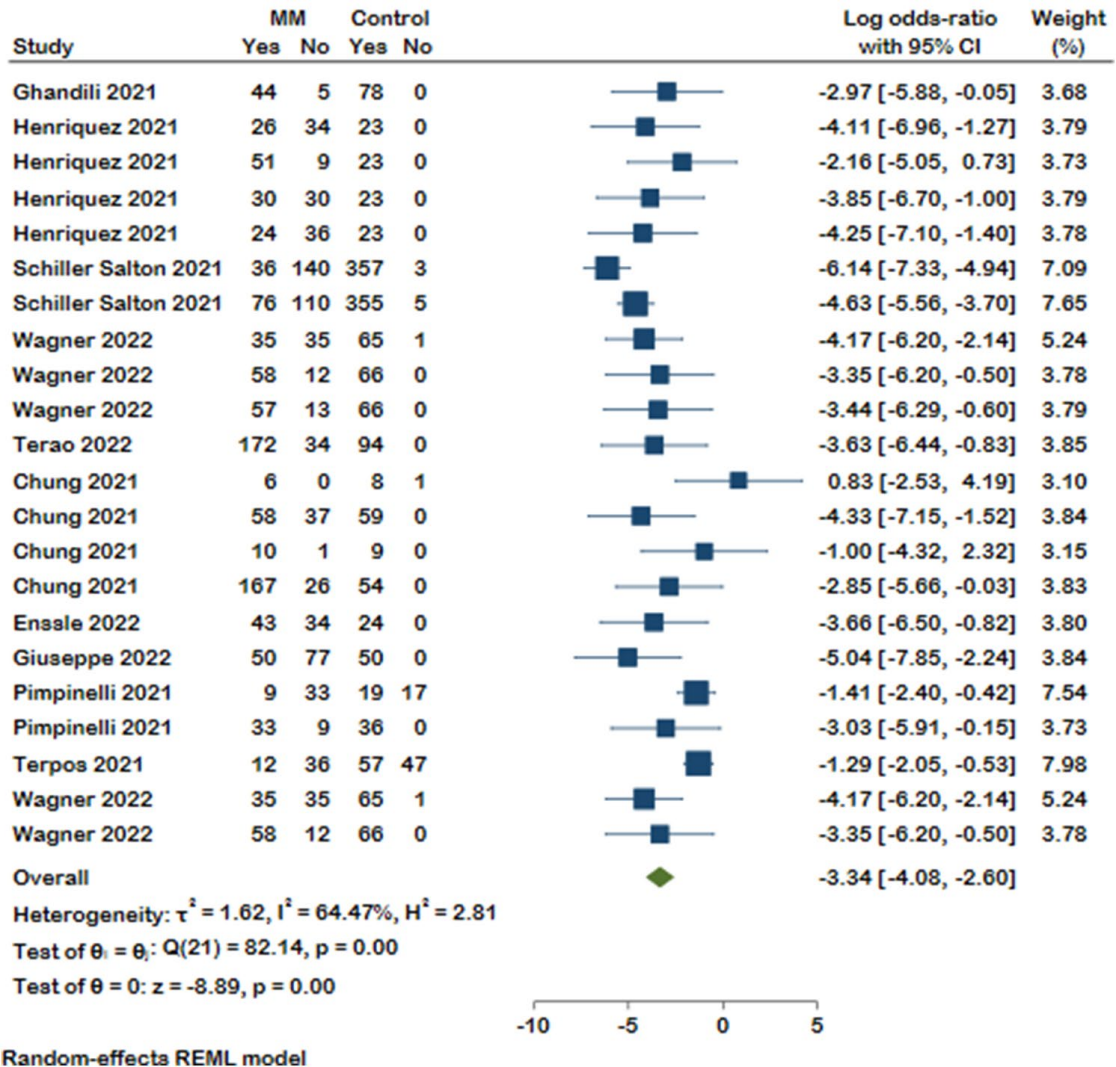
Forest plot of antibody responses in MM patients and controls

**Fig. 3:**
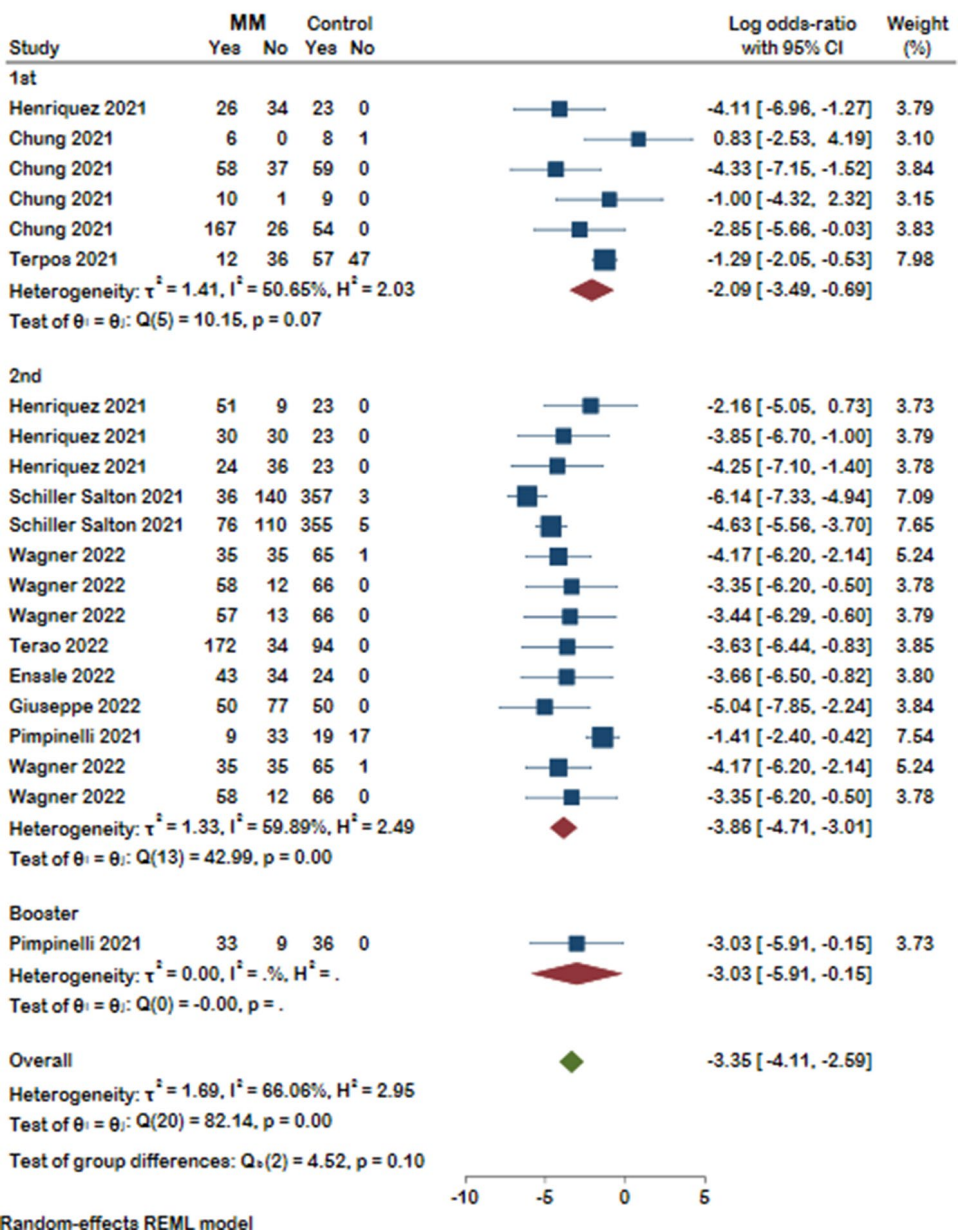
Forest plot of antibody responses in MM patients and controls categorized based on the number of the doses received

**Fig. 4:**
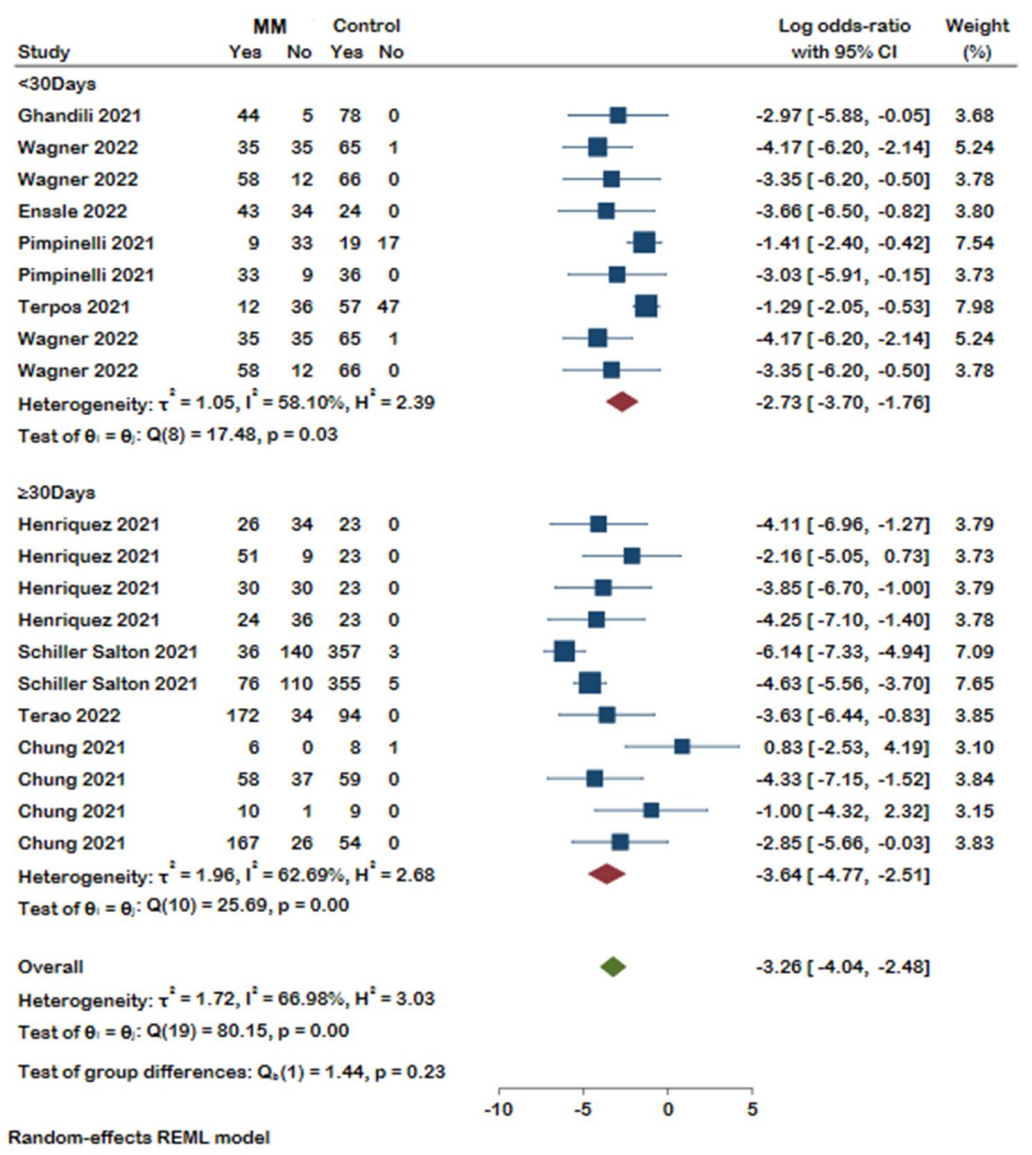
Forest plot of antibody responses in MM patients and controls categorized based on the time passed since their vaccination

**Fig. 5:**
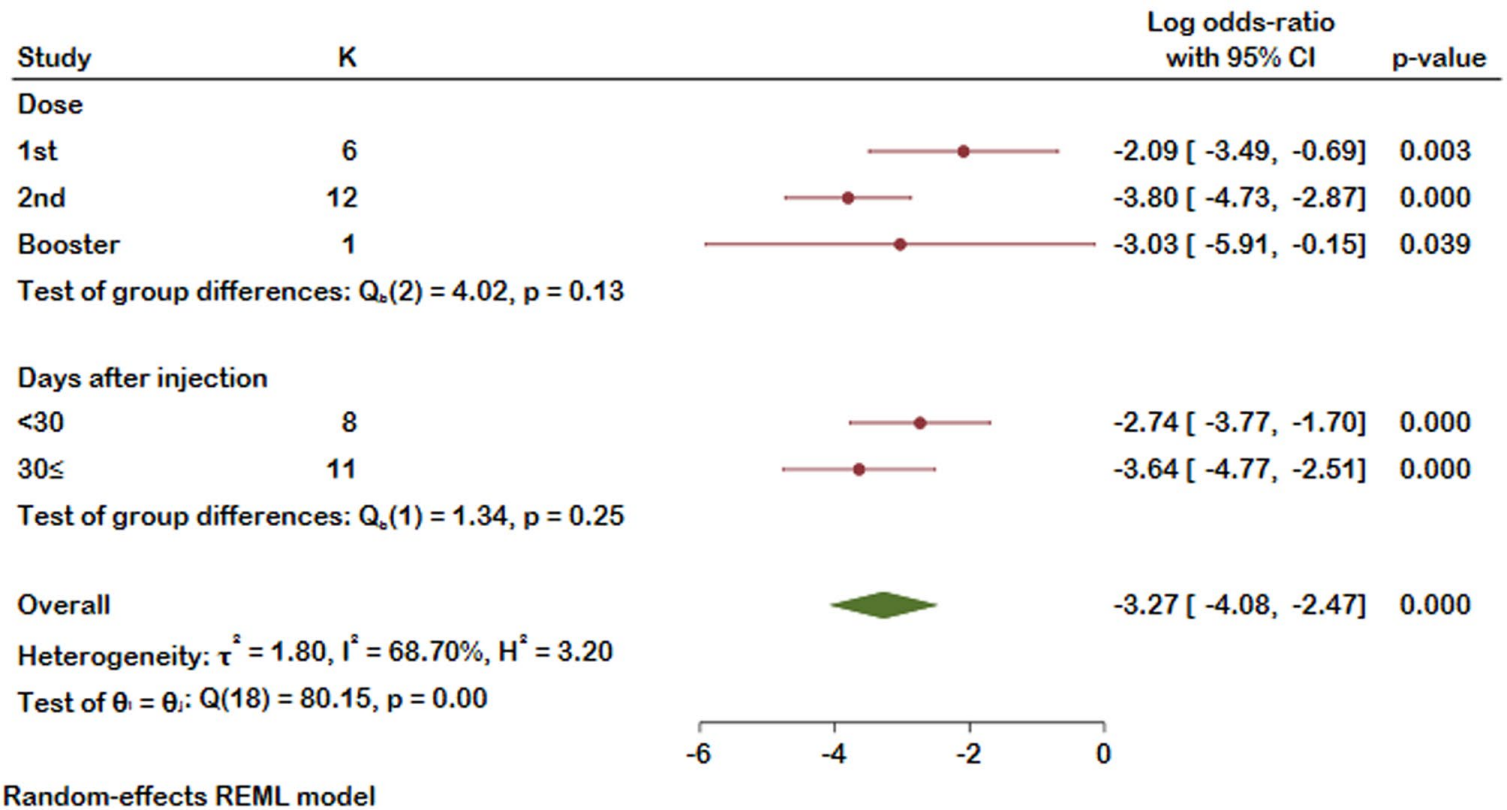
Summarized Forest plot of antibody responses in MM patients and controls based on their vaccination dose and time since vaccination. K represent the number of the studies for each variable

Our selected studies evaluated humoral response after COVID-19 vaccination by measuring SARS-CoV-2 spike IgG antibody (anti-S) or neutralizing antibody (nAb). Thirty-three studies reported antibody response, of which the detected antibody level was reported in sixteen studies. Our meta-analysis on eleven studies revealed that, compared with healthy controls, patients suffering from MM were less likely to form detectable antibodies after COVID-19 vaccination (Log odds ratio with 95% CI: -3.34 [-4.08, -2.60]), and substantial heterogeneity was identified (I²=64.47%). It should be noted that antibody response was evaluated at different time spans in six of the analyzed studies. Detailed results with the number of antibody responses in MM patients and controls are presented in the forest plot (Fig. 2). A distinct publication bias was detected, revealing an asymmetry in the funnel plot (Supplementary Fig. 1). As shown in Fig. 3 MM patients were less seropositive compared to healthy controls after first (log odds ratio − 2.09, 95%CI [-3.49, -0.69], I^2^ = 50.65%), second (log odds ratio − 3.80, 95%CI [-4.71, -3.01], I^2^ = 59.89%), and a booster dose (log odds ratio − 3.03, 95%CI [-5.91, -0.15]). No significant difference was detected between the groups (*p* = 0.10). We then stratified the antibody response in these studies based on the timing of assessment after vaccination (< 30 days and ≥ 30 days). Likewise, seropositivity was lower in MM patients compared with healthy controls regardless of the time passed from the vaccination (Fig. 4). The analysis results of all subgroups are represented in Fig. 5.

#### Antibody titration

**Fig. 6:**
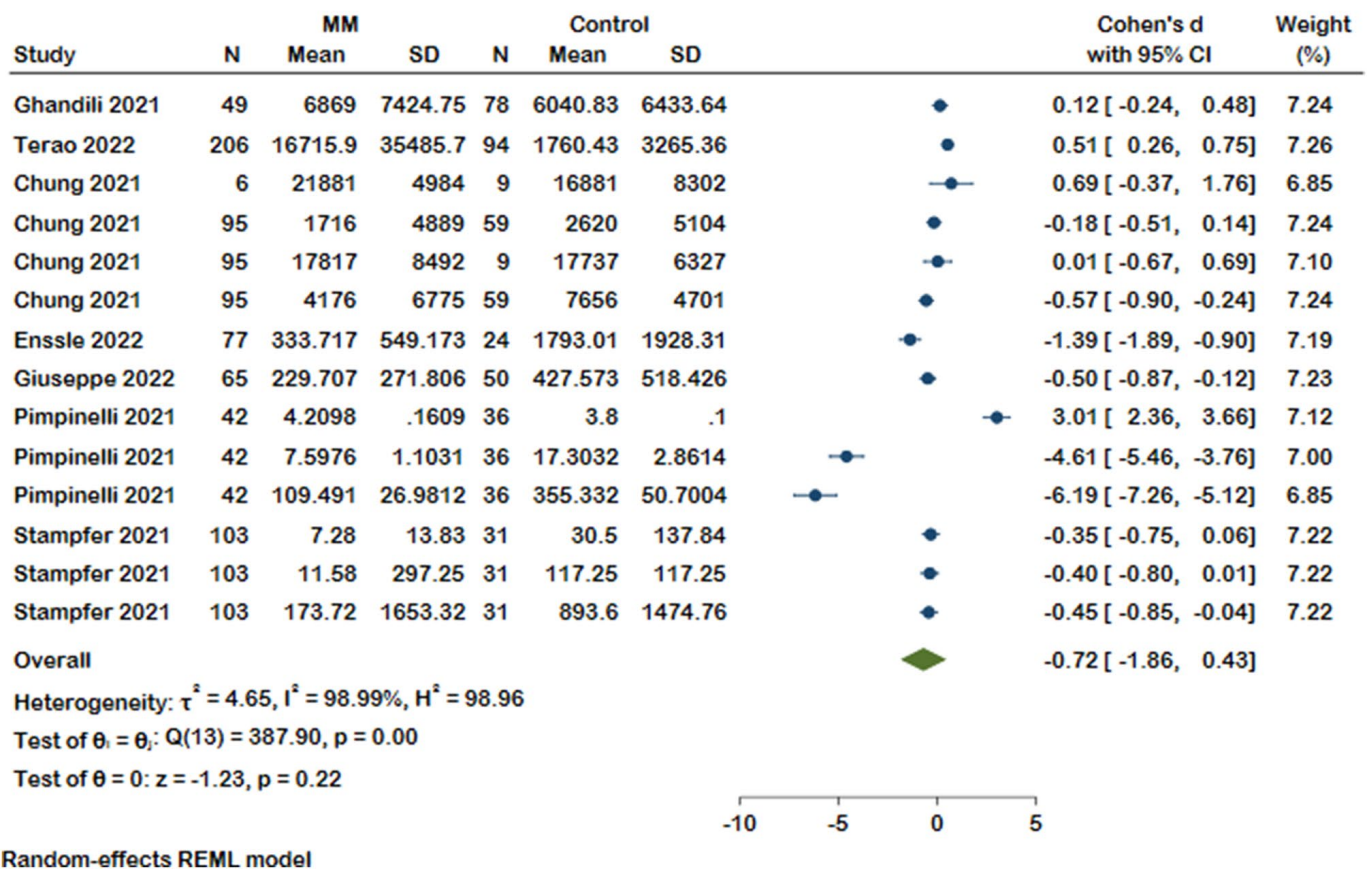
Forest plot of antibody titration results in MM patients and controls

Next, we focused our analysis on seven studies reporting the anti-S titration (Fig. 6). Interestingly, analysis showed no significant difference in mean level of anti-S between MM patients and healthy controls (Cohen’s d -0.72, 95% CI [-1.86, 0.43]), and a high level of heterogeneity (I^2^ = 98.99%). The high degree of heterogeneity in response to COVID-19 vaccination could be due to differences in the population, laboratory kits, measurement methods, and timing of titration after vaccination. In addition, an evidence of publication bias was noted in this analysis (Supplementary Fig. 2).

### T-cell responses

Only six studies evaluated T-cell-mediated immunity after COVID-19 vaccination (Table 2). Henriquez et al. measured IFN-γ production by T-cells after ex vivo stimulation with S1 or S2, three months after the first dose of BNT162b2 in MM patients and healthy controls. The IFN-γ was significantly lower in patients with MM compared with the controls [40]. In the study of Storti et al., vaccine-induced T cell response (spike-specific CD4 + or CD8 + T cell producing at least one of the three cytokines of IL-2 or IFN-γ or TNF-α) in MM patients and pre-malignant-monoclonal-gammopathies was evaluated by flow cytometry. Their results indicated that MM patients had a reduced T-cell response to complete vaccination and showed less cytotoxic IFN-γ + and TNF-α + CD8 + T cells than monoclonal gammopathy of undetermined significance (MGUS). Additionally, booster immunization improved cellular response to COVID-19 vaccination in both newly diagnosed MM (MMD) patients and relapsed/refractory MM (MMR) patients [41]. Wagner et al. analyzed the cellular response in 102 subjects, including healthy controls, IBD patients, MM patients, and patients with solid tumors. After the second dose, controls had a clear T-cell response upon stimulation with the S1 subunit. T cells of solid tumor patients secreted IL-2, IFN-γ, IL-17a and GM-CSF, and IL-10, while only IFN-γ, IL-17a, and IL-10 were induced in MM patients’ T-cells. IFN-γ and IL-2 were positively correlated to humoral response in controls and IBD patients, whereas only IL-2 was associated with antibody level in MM patients and patients with solid tumors [42]. In the study of Enssle et al. MM patients showed a diminished T-cell response after stimulation with the receptor-binding domain (RBD), the S2-protein, and CEF/CEFT control peptides following two doses of SARS-CoV-2 vaccination compared with controls. Lower frequencies of IFN-γ or interleukin-2 secreting CD4 + T cells were observed, whereas CD8 + T-cells did not differ between MM patients and the controls after stimulation [43]. Although CD4 + T cells were significantly lower in serological non-responders after the second vaccination, no positive correlation was observed between T-cell and serologic responses in patients with MM. Interestingly, T-cell response among serological responders and non-responders did not differ significantly [43]. Later, Enssle et al., evaluated variant specific T-cell response after the third dose of COVID-19 vaccination. Their results showed that patients with MM had a strong CD4^+^ T cell response against the (wild type) WT strain, while immune responses against Omicron was lower than the controls [44]. Zaleska et al. demonstrated specific SARS-CoV‐2 cytotoxic T-cells to evaluate immune response after mRNA vaccines in patients with hematologic malignancies. They found CD8 + T‐cell immune responses against SARS‐CoV‐2 spike epitope (YLQPRTFLL) in all HLA‐A*02 positive MM (*n* = 17). Notably, they reported a negative correlation between YLQPRTFLL‐specific CD8 + T-cells and antibody response [45]. Additionally, in MM patients, an increase in EM‐specific CD8 + T cells shortly after vaccination was observed, which decreased after 12 weeks [45].

**Table 2:** T-cell response in patients with Multiple myeloma after COVID-19 vaccination

Author/Year	Country	Study Design	MM no	Controls	Timing	Findings
Henriquez, [40]	France	Cohort	26	21	3 months after the first dose of BNT162b2	The IFN-γ production by T cells after stimulation with S1 or S2 antigens ex vivo was significantly lower in patients with MM.
Storti, [41]	Italy	Cohort	16	24	14 days after the second dose and 14 days after the booster	less cytotoxic IFN-γ + and TNF-α + CD8 + T cells in MM patients than MGUS
Wagner, [42]	Austria	Case Control	NA	NA	before the first dose and seven days after the second dose	IL-2 was associated with antibody level in MM patients
Enssle, [43]	Germany	Cohort	38	14	after 2 doses of vaccination	Lower frequencies of IFN-γ or interleukin-2 secreting CD4 + T cells were observed. CD8 + T-cells did not differ between MM patients and the controls.
Enssle, [43]	Germany	Cohort	71	23	1–3 months after the booster vaccination	MM patients had a strong CD4^+^ T cell response against the (wild type) WT strain. Immune responses against Omicron was lower than the controls.
Zaleska, [45]	Poland	Cohort	60	62	5 and 12 weeks after second dose	CD8 + T-cell immune responses against SARS‐CoV‐2 spike epitope (YLQPRTFLL) was found in all HLA‐A*02 positive MM patients.

MM: multiple myeloma; MGUS: monoclonal gammopathy of undetermined significance

### Clinical response

Seven of evaluated studies reported clinical response among vaccinated MM patients. In the study of Fillmore et al. on 1606 vaccinated MM patients and matched 1606 unvaccinated MM patients, among the vaccinated group, 14 (0.87%) were infected with COVID-19 following vaccination. In this matched cohort, vaccine effectiveness in MM patients was 22.2%, 14 days after the second dose [46]. In the study of Ghandili et al. of 82 vaccinated MM patients, one patient developed COVID-19 25 days after her first vaccination. At this time, the patient had no detectable antibody, while after infection, she was positive for nucleocapsid and spike antibodies [47]. Among a cohort of 60 MM patients with no history of SARS-CoV-2 infection, four were infected after one dose (*n* = 2) or two doses (*n* = 2) of BNT162b2, and all of them were receiving anti-CD38 immunotherapy [40]. During the prospective study of Ntanasis-Stathopoulos et al., between the third and fourth doses of BNT162b2, 34 (16.9%) were COVID-19 positive. The demographics and antibody levels of COVID-19-positive and negative patients were compared, and they had similar characteristics [48]. In a study by Schiller Salton, six months after the second vaccination, four of them186 patients developed COVID-19 at least one week following the latest vaccine dose (1 died, 1 had a severe disease but recovered, and 2 had a mild disease). Two patients didn’t have a serological response one month after the second vaccination, which was converted after their COVID‐19 resolution. One patient had a positive antibody response one month after vaccination, and his disease was mild [49]. Storti et al. monitored 40 patients with MM, SMM, or MGUS after the booster dose for about four months. The clinical follow-up didn’t show any disease progression. Three patients developed COVID-19 infections [41]. In a study conducted by Wang et al., MM patients’ risk for breakthrough infections after complete vaccination was 17.4% higher than the 4.5% in vaccinated patients without cancer. The risks for breakthrough infection did not differ based on race or ethnicity [50].

## Discussion and conclusions

To comprehensively evaluate the clinical response of COVID vaccines, it is insufficient to solely assess the B-cell and antibody response, as the crucial involvement of T-cell response in the body’s defense against the disease must also be considered [51, 52]. In contrast to the valuable findings of Gagelmann et al. that reported a pooled antibody response of 76% (95% CI: 67–83; I²=92%) in MM patients, our study endeavors to provide a more comprehensive analysis by systematically assessing two additional components of vaccine response in this patient population [13]. Besides, in order to ensure inclusivity and minimize data gaps, we aimed to incorporate all available studies in which the proportion of MM patients exceeded 85%. As depicted in the Supplementary Tables 3&4 showcasing our meta-regression analysis, there was no discernible difference in the outcome variable between studies solely comprising MM patients and those including 15% or less of other PCDs. Also, the results indicate that vaccine type and antibody measurement time did not significantly influence the observed outcomes, as evidenced by the lack of meaningful differences in the meta-regression analysis.

Different methodologies were employed by various studies to evaluate the impact of MM on the B-cell response, with seven studies using the mean and standard deviation of the antibody concentration in both case and control groups, while eleven studies measured the number of antibody positive and negative patients in each group. Our analysis incorporated both sets of studies, with results indicating a significant decrease in antibody concentration in the MM group as compared to the healthy control group (*p* = 0.00) in studies that utilized the mean and standard deviation method, as well as in studies that counted the number of antibody positive patients (*p* = 0.00). The subgroup analysis of the AB positive and negative studies based on the dosage of the vaccine demonstrates that the magnitude of the impact, as measured by the log odds ratio, was strongest for the second dose (-3.804), followed by the first dose (-2.091) and then the booster dose (-3.030). However, it is important to note that the sample size for the booster dose subgroup was only one study, which may limit the reliability of this finding (Supplementary Table 5).

The findings of a recent meta-analysis parallel our study [53], highlighting a diminished immune response among patients with MM following COVID-19 vaccination. However, our study differs in several significant respects. Notably, we integrated healthy controls into our analysis, serving as a comparative reference point. Furthermore, we included more studies in our analysis. Additionally, in addition to assessing B-cell and T-cell responses, we expanded our investigation to include evaluations of clinical responses.

Because of the compromised immune system in the disease which is exacerbated by immunosuppressive therapies, individuals with hematological malignancies are more likely to develop reduced vaccine response. Over the years, increasing evidence has revealed significant dysfunction of the immune system in individuals with MM [54, 55]. Consequently, both B-cell precursors and normal plasma cells are compromised, leading to consistent immune deficiency in patients with MM [56]. Additionally, despite an increase in effector cells like natural killer (NK) cells and cytotoxic CD8 + T cells in both bone marrow and peripheral blood, they fail to control disease progression, indicating a profound immunosuppressive environment [57]. Furthermore, dendritic cells exhibit alterations in MM, including diminished expression of co-stimulatory molecules and impaired initiation of allogeneic T-cell responses [58].

To our knowledge, no comprehensive review has evaluated the T-cell and clinical response of MM patients after COVID-19 vaccination at once, despite the critical role that these structures play in vaccine response. Therefore, this systematic review aims to fill this gap in the literature and provide a comprehensive evaluation of the vaccine response in MM patients. Most of the studies showed weaker T-cell response alongside antibody response among MM patients. The results of the evaluated studies in clinical response section indicates varying rates of COVID-19 infections among vaccinated MM patients, ranging from 0.87 to 17.4%. Some patients who experienced breakthrough infections had no detectable antibodies, while others exhibited a delayed serological response. These findings underscore the importance of continuous monitoring and potential risk factors for breakthrough infections in MM patients despite vaccination, emphasizing the need for further research in this area.

### Electronic supplementary material

Below is the link to the electronic supplementary material.

Supplementary Material 1

Supplementary Material 2

Supplementary Material 3

Supplementary Material 4

Supplementary Material 5

Supplementary Material 6

Supplementary Material 7

Supplementary Material 8

Supplementary Material 9

## Data Availability

Data are available upon a request to the corresponding author.

## References

[1] Blimark C (2015). Multiple myeloma and infections: a population-based study on 9253 multiple myeloma patients. Haematologica.

[2] Gavriatopoulou M (2021). SARS-CoV-2 vaccines in patients with multiple myeloma. Hemasphere.

[3] Ludwig H, Meckl A, Engelhardt M (2021). Compliance with vaccination recommendations among patients with multiple myeloma: a Real World Experience. Hemasphere.

[4] Vijenthira A (2020). Outcomes of patients with hematologic malignancies and COVID-19: a systematic review and meta-analysis of 3377 patients. Blood.

[5] Robertson JD (2000). Immunogenicity of vaccination against influenza, Streptococcus pneumoniae and Haemophilus influenzae type B in patients with multiple myeloma. Br J Cancer.

[6] Hahn M (2015). Efficacy of single versus boost vaccination against influenza virus in patients with multiple myeloma. Haematologica.

[7] Dagnew AF (2019). Immunogenicity and safety of the adjuvanted recombinant zoster vaccine in adults with haematological malignancies: a phase 3, randomised, clinical trial and post-hoc efficacy analysis. Lancet Infect Dis.

[8] Terrault NA (2018). Update on prevention, diagnosis, and treatment of chronic hepatitis B: AASLD 2018 hepatitis B guidance. Hepatology.

[9] Ludwig H (2021). Recommendations for vaccination in multiple myeloma: a consensus of the European Myeloma Network. Leukemia.

[10] Geno KA (2015). Pneumococcal capsules and their types: past, Present, and Future. Clin Microbiol Rev.

[11] Palazzo M (2018). Revaccination after autologous hematopoietic stem cell transplantation is safe and effective in patients with multiple myeloma receiving Lenalidomide maintenance. Biol Blood Marrow Transpl.

[12] Rieger CT (2018). Anti-infective vaccination strategies in patients with hematologic malignancies or solid tumors-Guideline of the Infectious Diseases Working Party (AGIHO) of the German Society for Hematology and Medical Oncology (DGHO). Ann Oncol.

[13] Gagelmann N (2022). Antibody response after vaccination against SARS-CoV-2 in adults with hematological malignancies: a systematic review and meta-analysis. Haematologica.

[14] Ljungman P (2021). COVID-19 and stem cell transplantation; results from an EBMT and GETH multicenter prospective survey. Leukemia.

[15] Newcastle-. Ottawa scale (NOS), available online at: https://www.ohri.ca/programs/clinical_epidemiology/nosgen.pdf, accessed: April 4th, 2023.

[16] Abdallah AO, Mahmoudjafari Z, Atieh T, Ahmed N, Cui W, Shune L, et al. Neutralizing antibody responses against SARS-CoV-2 in patients with plasma cell disorders who are on active treatment after two doses of mRNA vaccination. Eur J Haematol. 2022;109(5):458–64. 10.1111/ejh.13826. 10.1111/ejh.13826 PMC9350358 35810359

[17] Abella E, Trigueros M, Pradenas E, Muñoz-Lopez F, Garcia-Pallarols F, Ben Azaiz Ben Lahsen R, et al. Efficacy of SARS-CoV-2 vaccination in patients with monoclonal gammopathies: a cross sectional study. Life Sci Alliance. 2022;5(12):e202201479. 10.26508/lsa.202201479. 10.26508/lsa.202201479 PMC9375155 35961779

[18] Bird S, Panopoulou A, Shea RL, Tsui M, Saso R, Sud A, et al. Response to first vaccination against SARS-CoV-2 in patients with multiple myeloma. Lancet Haematol. 2021;8(6):e389–e392. 10.1016/S2352-3026(21)00110-1. 10.1016/S2352-3026(21)00110-1 PMC8055205 33887255

[19] Bitoun S, Henry J, Vauloup-Fellous C, Dib N, Belkhir R, Mouna L, et al. Response to COVID-19 mRNA vaccination in multiple myeloma is conserved but impaired compared to controls. J Hematol Oncol. 2021;14(1):166. 10.1186/s13045-021-01183-2. 10.1186/s13045-021-01183-2 PMC8512646 34645504

[20] Chan WY, Howells L, Wilson W, Sanchez E, Ainley L, Chavda SJ, et al. Serological response to the BNT162b2 mRNA or ChAdOx1 nCoV-19 COVID-19 vaccine after first and second doses in patients with plasma cell disorders: influence of host and disease factors. Br J Haematol. 2022;196(3):e21–e26. 10.1111/bjh.17864. 10.1111/bjh.17864 PMC8652995 34632575

[21] Chung DJ, Shah GL, Devlin SM, Ramanathan LV, Doddi S, Pessin MS, et al. Disease- and therapy-specific impact on humoral immune responses to COVID-19 vaccination in hematologic malignancies. Blood Cancer Discov. 2021;2(6):568–76. 10.1158/2643-3230.BCD-21-0139. 10.1158/2643-3230.BCD-21-0139 PMC8580617 34778797

[22] Fattizzo B, Bortolotti M, Rampi N, Cavallaro F, Giannotta JA, Bucelli C, et al. Seroconversion to mRNA SARS-CoV-2 vaccines in hematologic patients. Front Immunol. 2022;13:852158. 10.3389/fimmu.2022.852158. 10.3389/fimmu.2022.852158 PMC9133475 35634287

[23] Ghandili S, Schönlein M, Wiessner C, Becher H, Lütgehetmann M, Brehm TT, et al. Lymphocytopenia and anti-CD38 directed treatment impact the serological SARS-CoV-2 response after prime boost vaccination in patients with multiple myeloma. J Clin Med. 2021;10(23):5499. 10.3390/jcm10235499. 10.3390/jcm10235499 PMC8658197 34884200

[24] Giuseppe M, Claudia R, Antonella M, Angelo S, Domenico P. Long follow-up of symptomatic multiple myeloma patients after Covid-19 vaccination (BNT162b2): a single-institution retrospective experience. Leuk Res Rep. 2022;18:100342. 10.1016/j.lrr.2022.100342. 10.1016/j.lrr.2022.100342 PMC9359603 35966626

[25] Greenberg RS, Ruddy JA, Boyarsky BJ, Werbel WA, Garonzik-Wang JM, Segev DL, et al. Safety and antibody response to two-dose SARS-CoV-2 messenger RNA vaccination in patients with multiple myeloma. BMC Cancer. 2021 Dec 27;21(1):1354. 10.1186/s12885-021-09097-5. 10.1186/s12885-021-09097-5 PMC8711688 34961488

[26] Gung C, McGuire R, George M, Abdulkareem A, Belden KA, Porcu P, et al. Antibody response to SARS-CoV-2 vaccination in patients with lymphoproliferative disorders and plasma cell dyscrasias: anti-lymphoma therapy as a predictive biomarker of response to vaccination. Front Oncol. 2022;12:840451. 10.3389/fonc.2022.840451. 10.3389/fonc.2022.840451 PMC9300919 35875166

[27] Haggenburg S, Hofsink Q, Lissenberg-Witte BI, Broers AEC, van Doesum JA, van Binnendijk RS, et al. Antibody response in immunocompromised patients with hematologic cancers who received a 3-dose mRNA-1273 vaccination schedule for COVID-19. JAMA Oncol. 2022;8(10):1477–483. 10.1001/jamaoncol.2022.3227. 10.1001/jamaoncol.2022.3227 PMC9372904 35951338

[28] Haggenburg S, Lissenberg-Witte BI, van Binnendijk RS, den Hartog G, Bhoekhan MS, Haverkate NJE, et al. Quantitative analysis of mRNA-1273 COVID-19 vaccination response in immunocompromised adult hematology patients. Blood Adv. 2022;6(5):1537–546. 10.1182/bloodadvances.2021006917. 10.1182/bloodadvances.2021006917 PMC8816838 35114690

[29] Hallmeyer S, Thompson MA, Fitzpatrick V, Liao Y, Mullane MP, Medlin SC, et al. Characteristics of patients with hematologic malignancies without seroconversion post-COVID-19 third vaccine dosing. Biol Methods Protoc. 2023;8(1):bpad002. 10.1093/biomethods/bpad002. 10.1093/biomethods/bpad002 PMC9982360 36873569

[30] Marasco V, Carniti C, Guidetti A, Farina L, Magni M, Miceli R, et al. T-cell immune response after mRNA SARS-CoV-2 vaccines is frequently detected also in the absence of seroconversion in patients with lymphoid malignancies. Br J Haematol. 2022;196(3):548–58. 10.1111/bjh.17877. 10.1111/bjh.17877 PMC8653177 34649298

[31] Nooka AK, Shanmugasundaram U, Cheedarla N, Verkerke H, Edara VV, Valanparambil R, et al. Determinants of neutralizing antibody response after SARS CoV-2 vaccination in patients with myeloma. J Clin Oncol. 2022;40(26):3057–3064. 10.1200/JCO.21.02257. 10.1200/JCO.21.02257 PMC9462534 35259002

[32] Pimpinelli F, Marchesi F, Piaggio G, Giannarelli D, Papa E, Falcucci P, et al. Fifth-week immunogenicity and safety of anti-SARS-CoV-2 BNT162b2 vaccine in patients with multiple myeloma and myeloproliferative malignancies on active treatment: preliminary data from a single institution. J Hematol Oncol. 2021;14(1):81. 10.1186/s13045-021-01090-6. 10.1186/s13045-021-01090-6 PMC8128283 34001183

[33] Ramasamy K, Sadler R, Jeans S, Varghese S, Turner A, Larham J, et al. COVID symptoms, testing, shielding impact on patient-reported outcomes and early vaccine responses in individuals with multiple myeloma. Br J Haematol. 2022;196(1):95–98. 10.1111/bjh.17764. 10.1111/bjh.17764 PMC8444854 34341984

[34] Re D, Seitz-Polski B, Brglez V, Carles M, Graça D, Benzaken S, et al. Humoral and cellular responses after a third dose of SARS-CoV-2 BNT162b2 vaccine in patients with lymphoid malignancies. Nat Commun. 2022;13(1):864. 10.1038/s41467-022-28578-0. 10.1038/s41467-022-28578-0 PMC8844396 35165284

[35] Stampfer SD, Goldwater MS, Jew S, Bujarski S, Regidor B, Daniely D, et al. Response to mRNA vaccination for COVID-19 among patients with multiple myeloma. Leukemia. 2021;35(12):3534–541. 10.1038/s41375-021-01354-7. 10.1038/s41375-021-01354-7 PMC8320411 34326466

[36] Terao T, Yamashita T, Fukumoto A, Kamura Y, Ikeda D, Kuzume A, et al. Low clinical protective response to SARS-CoV-2 mRNA COVID-19 vaccine in patients with multiple myeloma. Int J Hematol. 2022;115(5):737–47. 10.1007/s12185-022-03300-4. 10.1007/s12185-022-03300-4 PMC8860256 35190963

[37] Terao T, Naduka T, Ikeda D, Fukumoto A, Kamura Y, Kuzume A, et al. Depletion of CD38-positive regulatory T cells by anti-CD38 monoclonal antibodies induces a durable response to SARS-CoV-2 vaccination in patients with plasma cell dyscrasia. Br J Haematol. 2022;197(4):417–21. 10.1111/bjh.18079. 10.1111/bjh.18079 PMC9111412 35172374

[38] Terpos E, Trougakos IP, Gavriatopoulou M, Papassotiriou I, Sklirou AD, Ntanasis-Stathopoulos I, et al. Low neutralizing antibody responses against SARS-CoV-2 in older patients with myeloma after the first BNT162b2 vaccine dose. Blood. 2021;137(26):3674–676. 10.1182/blood.2021011904. 10.1182/blood.2021011904 PMC8061093 33861315

[39] Thompson MA, Hallmeyer S, Fitzpatrick VE, Liao Y, Mullane MP, Medlin SC, et al. Real-world third COVID-19 vaccine dosing and antibody response in patients with hematologic malignancies. J Patient Cent Res Rev. 2022;9(3):149–57. 10.17294/2330-0698.1952. 10.17294/2330-0698.1952 PMC9302908 35935520

[40] Henriquez S (2022). Anti-CD38 therapy impairs SARS-CoV-2 vaccine response against alpha and delta variants in patients with multiple myeloma. Blood.

[41] Storti P et al. Impact of omicron variant on the response to sars-cov-2 mrna vaccination in multiple myeloma. 2022. 10.1080/2162402X.2022.2120275 PMC9467550 36105747

[42] Wagner A (2022). SARS-CoV-2-mRNA booster vaccination reverses non-responsiveness and early antibody waning in Immunocompromised patients - A Phase Four Study comparing Immune responses in patients with solid cancers, multiple myeloma and inflammatory bowel disease. Front Immunol.

[43] Enssle JC (2022). Enhanced but variant-dependent serological and cellular immune responses to third-dose BNT162b2 vaccination in patients with multiple myeloma. Cancer Cell.

[44] Enssle JC (2022). Severe impairment of T-cell responses to BNT162b2 immunization in patients with multiple myeloma. Blood.

[45] Zaleska J et al. Response to anti-SARS-CoV-2 mRNA vaccines in multiple myeloma and chronic lymphocytic leukemia patients. Int J Cancer, 2022. 10.1002/ijc.34209 PMC9349960 35830214

[46] Fillmore NR (2021). Inadequate sars-cov-2 vaccine effectiveness in patients with multiple myeloma: a large nationwide veterans affairs study. Blood.

[47] Ghandili S et al. Post-vaccination Anti-SARS-CoV-2-Antibody response in patients with multiple myeloma correlates with low CD19 + B-Lymphocyte Count and Anti-CD38 Treatment. Cancers (Basel), 2021; 13(15). 10.3390/cancers13153800 PMC8345197 34359701

[48] Ntanasis-Stathopoulos I (2022). Second booster BNT162b2 restores SARS-CoV-2 humoral response in patients with multiple myeloma, excluding those under Anti-BCMA therapy. Hemasphere.

[49] Schiller Salton N (2021). Attenuated humoral immune response following anti-SARS-CoV-2 vaccine in heavily pretreated patients with multiple myeloma and AL amyloidosis. Am J Hematol.

[50] Wang L (2022). COVID-19 breakthrough infections, hospitalizations and mortality in fully vaccinated patients with hematologic malignancies: a clarion call for maintaining mitigation and ramping-up research. Blood Rev.

[51] Chen Y (2022). Dynamic SARS-CoV-2-specific B-cell and T-cell responses following immunization with an inactivated COVID-19 vaccine. Clin Microbiol Infect.

[52] Keppler-Hafkemeyer A (2023). Potent high-avidity neutralizing antibodies and T cell responses after COVID-19 vaccination in individuals with B cell lymphoma and multiple myeloma. Nat Cancer.

[53] Chuleerarux N (2022). Immunogenicity of SARS-CoV-2 vaccines in patients with multiple myeloma: a systematic review and meta-analysis. Blood Adv.

[54] Pratt G, Goodyear O, Moss P (2007). Immunodeficiency and immunotherapy in multiple myeloma. Br J Haematol.

[55] Noonan K, Borrello I (2011). The immune microenvironment of myeloma. Cancer Microenviron.

[56] Rawstron AC (1998). B-lymphocyte suppression in multiple myeloma is a reversible phenomenon specific to normal B-cell progenitors and plasma cell precursors. Br J Haematol.

[57] Pérez-Andres M (2006). Characterization of bone marrow T cells in monoclonal gammopathy of undetermined significance, multiple myeloma, and plasma cell leukemia demonstrates increased infiltration by cytotoxic/Th1 T cells demonstrating a squed TCR-Vbeta repertoire. Cancer.

[58] Ratta M (2002). Dendritic cells are functionally defective in multiple myeloma: the role of interleukin-6. Blood.

