# Supplementary material for: Examining the immunological responses to COVID-19 vaccination in multiple myeloma patients: a systematic review and meta-analysis
Source: BMC Geriatr. 2024 May 8;24:411. doi: 10.1186/s12877-024-05006-0 (PMC11080142; doi:10.1186/s12877-024-05006-0)
Supplement: Supplementary file 1 — Supplementary Material 1 [file 12877_2024_5006_MOESM1_ESM.docx]

**Table S1.** Search term for each database

| **1. Pubmed:** 72  (((((((((Multiple myeloma[Title/Abstract]) OR (Plasma cell myeloma[Title/Abstract])) OR (Plasma cell dyscrasia[Title/Abstract])) OR (Myeloma multiplex[Title/Abstract])) OR (Myelomatosis[Title/Abstract])) OR (Kahler disease[Title/Abstract])) OR (Kahler’s disease[Title/Abstract])) OR (Morbus kahler[Title/Abstract])) AND (((((COVID-19[Title/Abstract]) OR (SARS-CoV-2[Title/Abstract])) OR (SARS-CoV2[Title/Abstract])) OR (2019-nCoV[Title/Abstract])) OR (Novel coronavirus[Title/Abstract]))) AND (((((Vaccine*[Title/Abstract]) OR (Vaccination[Title/Abstract])) OR (Immunization[Title/Abstract])) OR (Active immunization[Title/Abstract])) OR (Vaccinated[Title/Abstract])) |
| --- |
| **2. Embase:** 136  ('multiple myeloma':ab,ti OR 'plasma cell myeloma':ab,ti OR 'plasma cell dyscrasia':ab,ti OR 'myeloma multiplex':ab,ti OR 'myelomatosis':ab,ti OR 'kahler disease':ab,ti OR 'morbus kahler':ab,ti) AND ('covid-19':ti,ab,kw OR 'sars-cov-2':ti,ab,kw OR 'sars-cov2':ti,ab,kw OR '2019-ncov':ti,ab,kw OR 'novel coronavirus':ti,ab,kw) AND ('vaccine*':ti,ab,kw OR 'vaccination':ti,ab,kw OR 'immunization':ti,ab,kw OR 'active immunization':ti,ab,kw OR 'vaccinated':ti,ab,kw) |
| **3. Web of science:** 75  TOPIC: "Multiple myeloma" OR "Plasma cell myeloma" OR "Plasma cell dyscrasia" OR "Myeloma multiplex" OR "Myelomatosis" OR "Kahler disease" OR "Kahler's disease" OR "Morbus kahler") AND TOPIC: ("COVID-19" OR "SARS-CoV-2" OR "SARS-CoV2" OR "2019-nCoV" OR "Novel coronavirus") AND TOPIC: ("Vaccine*" OR "Vaccination" OR "Immunization" OR "Active immunization" OR "Vaccinated") Timespan: All years. Indexes: SCI-EXPANDED, SSCI, A&HCI, CPCI-S, CPCI-SSH, BKCI-S, BKCI-SSH, ESCI, CCR-EXPANDED, IC. |
| **4. Cochrane:** 5  #1: (Multiple Myeloma):ti,ab,kw OR (Plasma cell myeloma):ti,ab,kw OR (Plasma cell dyscrasia):ti,ab,kw OR (Kahler disease):ti,ab,kw OR (Myelomatosis):ti,ab,kw  #2: (COVID-19):ti,ab,kw OR (SARS-CoV-2):ti,ab,kw OR (SARS-CoV2):ti,ab,kw OR (Novel coronavirus):ti,ab,kw  #3: (Vaccine*):ti,ab,kw OR (Vaccination):ti,ab,kw OR (Immunization):ti,ab,kw OR (Active Immunization):ti,ab,kw OR (Vaccinated):ti,ab,kw  #4: #1 AND #2 AND #3 |
| **5. Other sources (searching manually):** 7 |
