# Supplementary material for: Examining the immunological responses to COVID-19 vaccination in multiple myeloma patients: a systematic review and meta-analysis
Source: BMC Geriatr. 2024 May 8;24:411. doi: 10.1186/s12877-024-05006-0 (PMC11080142; doi:10.1186/s12877-024-05006-0)
Supplement: Supplementary file 2 — Supplementary Material 2 [file 12877_2024_5006_MOESM2_ESM.docx]

**Table S2.** Newcastle-Ottawa scale (NOS) risk of bias assessment of the included studies

|  | Selection | | | | | Comparability | | Exposure | | | |
| --- | --- | --- | --- | --- | --- | --- | --- | --- | --- | --- | --- |
| Study First author (year) | Case definition adequacy | Representative of cases | Selection of controls | Definition of controls | Subtotal | Comparability of cases and controls on the basis of the design or analysis | Subtotal | Ascertainment of exposure | Same method of ascertainment for cases and controls | Non-response rate | Subtotal |
| Abdallah (2022) |  |  | * | * | 2 | ** | 2 | * | * | * | 3 |
| Abella (2022) | * | * | * | * | 4 | * | 1 | * |  |  | 1 |
| Bird(2021) |  | * |  | * | 2 | ** | 2 | * | * | * | 3 |
| Bitoun(2012) |  | * |  | * | 2 | ** | 2 | * | * | * | 3 |
| Chan(2022) | * | * | * | * | 4 | ** | 2 | * |  |  | 1 |
| Chung (2021) |  | * |  | * | 2 | * | 1 | * | * | * | 3 |
| Enssle (2022) | * | * | * | * | 4 | * | 1 | * | * |  | 2 |
| Fattizzo (2022) |  | * | * | * | 3 | ** | 2 | * | * | * | 3 |
| Fillmore(2021) | * | * | * | * | 4 | * | 1 | * |  |  | 1 |
| Gaviratopoulou(2021) |  | * |  | * | 2 | ** | 2 | * | * | * | 3 |
| Ghandili (2021) |  | * | * |  | 2 | ** | 2 | * | * |  | 2 |
| Ghandili (2021) |  |  | * | * | 2 | ** | 2 | * | * | * | 3 |
| Giuseppe (2022) |  | * |  | * | 2 | ** | 2 | * | * | * | 3 |
| Greenberg(2021) |  | * |  |  | 1 | ** | 2 | * | * | * | 3 |
| Gung(2022) |  | * |  |  | 1 | ** | 2 | * | * | * | 3 |
| Haggenburg(2022) | * | * |  | * | 3 | ** | 2 | * | * | * | 3 |
| Haggenburg (2022) |  | * | * | * | 3 | * | 1 | * | * | * | 3 |
| Hallmeyer(2022) | * | * | * | * | 4 | ** | 2 | * | * | * | 3 |
| Henriquez(2021) |  |  | * | * | 2 | ** | 2 | * | * | * | 3 |
| Marasco (2022) |  |  | * | * | 2 | ** | 2 |  | * | * | 2 |
| Nooka(2022) | * | * |  |  | 2 | ** | 2 | * |  |  | 1 |
| Ntanasis-Stathopoulos(2022) | * | * | * | * | 4 | ** | 2 | * | * | * | 3 |
| Pimpinelli (2021) | * | * | * | * | 4 | ** | 2 | * |  |  | 1 |
| Ramasamy(2021) |  |  |  | * | 1 | * | 1 |  |  | * | 1 |
| Re(2022) |  |  | * | * | 2 | ** | 2 | * | * | * | 3 |
| Schiller Salton (2021) |  | * | * | * | 3 | ** | 2 | * | * | * | 3 |
| Storti(2022) |  | * | * | * | 3 | ** | 2 | * |  |  | 1 |
| Stampfer (2021) | * | * |  | * | 3 | ** | 2 |  | * | * | 2 |
| Terao (2022) | * | * | * |  | 3 | * | 1 |  | * | * | 2 |
| Terao (2022) |  |  | * | * | 2 | ** | 2 | * | * | * | 3 |
| Terpos (2021) | * | * | * | * | 4 | * | 1 | * | * | * | 3 |
| Thompson (2022) | * | * | * | * | 4 | ** | 2 | * |  |  | 1 |
| Wagner (2022) | * | * | * | * | 4 | * | 1 | * | * | * | 3 |
| Wang (2022) | * | * | * | * | 4 | * | 1 | * | * | * | 3 |
| Zaleska (2022) | * | * | * | * | 4 | * | 1 | * | * | * | 3 |
