# Supplementary material for: Examining the immunological responses to COVID-19 vaccination in multiple myeloma patients: a systematic review and meta-analysis
Source: BMC Geriatr. 2024 May 8;24:411. doi: 10.1186/s12877-024-05006-0 (PMC11080142; doi:10.1186/s12877-024-05006-0)
Supplement: Supplementary file 3 — Supplementary Material 3 [file 12877_2024_5006_MOESM3_ESM.docx]

**Table S3.** Random-effects meta-regression

| Factor | Coefficient | Std err | Z | P-value | 95% conf. interval |
| --- | --- | --- | --- | --- | --- |
| Time | -1.021009 | 0.8034771 | -1.27 | 0.204 | [-2.595796, 0.5537769] |
| Vaccine Type | 0.8594943 | 1.139765 | 0.75 | 0.451 | [-1.374404, 3.093392] |
| MM or PCD | -0.5007092 | 18 | 2.209774 | 0.821 | [-4.831787, 3.830369] |

**Table S4.** Residual heterogeneity

| Parameter | tau^2 | I^2 (%) | H^2 | R-squared (%) | Wald chi2(3) | Prob > chi2 |
| --- | --- | --- | --- | --- | --- | --- |
| Results | 1.54 | 62.86 | 2.69 | 10.23 | 1.91 | 0.5922 |
