# Supplementary material for: Examining the immunological responses to COVID-19 vaccination in multiple myeloma patients: a systematic review and meta-analysis
Source: BMC Geriatr. 2024 May 8;24:411. doi: 10.1186/s12877-024-05006-0 (PMC11080142; doi:10.1186/s12877-024-05006-0)
Supplement: Supplementary file 4 — Supplementary Material 4 [file 12877_2024_5006_MOESM4_ESM.docx]

**Table S5.** Meta-analysis results and subgroups

| Parameter | Subgroup | No. of contingency tables | Log odds-ratio | 95% conf.interval | P-value |
| --- | --- | --- | --- | --- | --- |
| Dose | 1 | 6 | -2.091 | [-3.488, -0.693] | 0.003 |
|  | 2 | 12 | -3.804 | [-4.734, -2.873] | 0.000 |
|  | 3 | 1 | -3.030 | [-5.912, -0.148] | 0.039 |
| Time | 30 Days < | 8 | -2.735 | [-3.772, -1.699] | 0.000 |
|  | ≤ 30 Days | 11 | -3.640 | [-4.766, -2.513] | 0.000 |
| Overall | - | 19 | -3.274 | [-4.081, -2.467] | 0.000 |
