# Supplementary material for: Examining the immunological responses to COVID-19 vaccination in multiple myeloma patients: a systematic review and meta-analysis
Source: BMC Geriatr. 2024 May 8;24:411. doi: 10.1186/s12877-024-05006-0 (PMC11080142; doi:10.1186/s12877-024-05006-0)
Supplement: Supplementary file 5 — Supplementary Material 5 [file 12877_2024_5006_MOESM5_ESM.docx]

**Table S6.** Heterogeneity summary

| Parameter | Subgroup | df | Q ( P- Value) | tau^2 | I^2 | H^2 |
| --- | --- | --- | --- | --- | --- | --- |
| Dose | 1 | 5 | 10.15  (0.071) | 1.408 | 50.65 | 2.03 |
|  | 2 | 11 | 42.20  (0.000) | 1.480 | 64.43 | 2.81 |
| Time | 30 Days < | 7 | 17.05  (0.017) | 1.166 | 62.52 | 2.67 |
|  | ≤ 30 Days | 10 | 25.69  (0.004) | 1.957 | 62.69 | 2.68 |
| Overall | - | 18 | 80.15  (0.000) | 1.799 | 68.70 | 3.20 |

**Table S7.** Tests of group differences

| Parameter | df | Q ( P- Value) |
| --- | --- | --- |
| Dose | 2 | 4.02  (0.134) |
| Timebin | 1 | 1.34  (0.247) |
