# Supplementary figures and images for: Examining the immunological responses to COVID-19 vaccination in multiple myeloma patients: a systematic review and meta-analysis
Source: BMC Geriatr. 2024 May 8;24:411. doi: 10.1186/s12877-024-05006-0 (PMC11080142; doi:10.1186/s12877-024-05006-0)

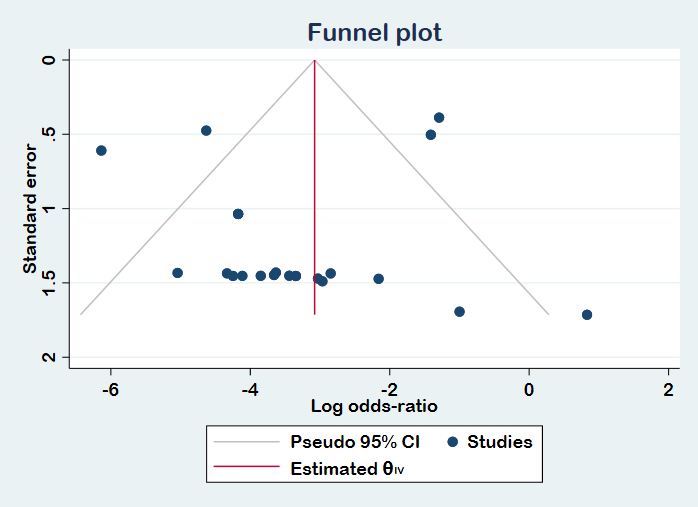

Supplement: Supplementary file 6 — Supplementary Material 6 [file 12877_2024_5006_MOESM6_ESM.tif]

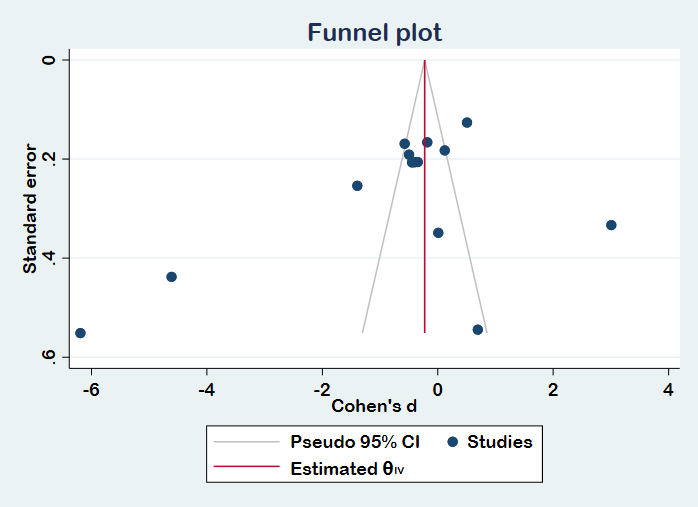

Supplement: Supplementary file 7 — Supplementary Material 7 [file 12877_2024_5006_MOESM7_ESM.tif]

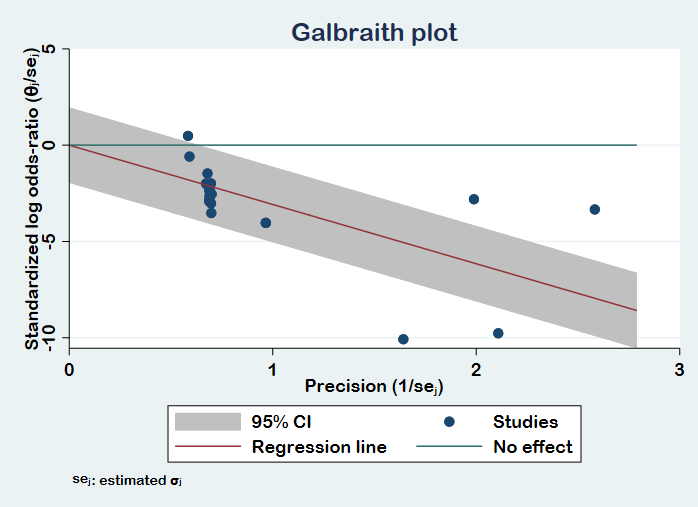

Supplement: Supplementary file 8 — Supplementary Material 8 [file 12877_2024_5006_MOESM8_ESM.tif]

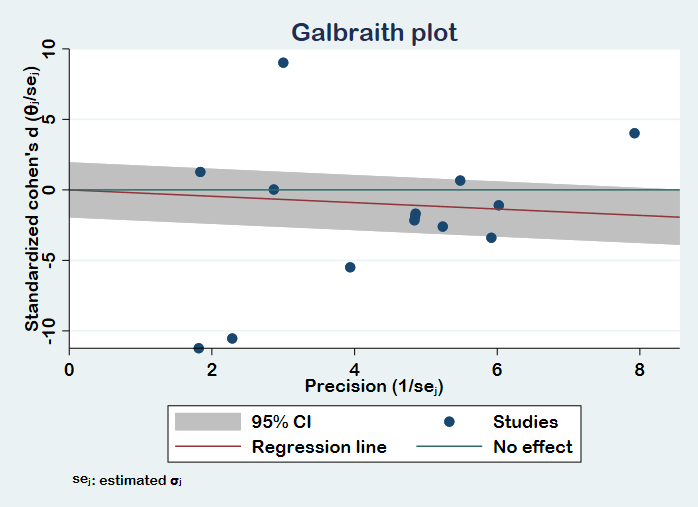

Supplement: Supplementary file 9 — Supplementary Material 9 [file 12877_2024_5006_MOESM9_ESM.tif]
